# Supplementary material for: Mutation Profile of Aggressive Pheochromocytoma and Paraganglioma with Comparison of TCGA Data
Source: Cancers (Basel). 2021 May 14;13(10):2389. doi: 10.3390/cancers13102389 (PMC8156611; doi:10.3390/cancers13102389)
Supplement: Supplementary file 1 [file cancers-13-02389-s001.zip › cancers-1185566-supplementary.pdf]

Supplementary Table S1. Gene List for Targeted Next-Generation Sequencing

ABCB1	BIRC2	CIC	ERBB2	FLT1	JAK3	MLL3	NTRK2	REL	SPECC1	VTI1B
ABCC2	BIRC3	CLP1	ERBB2IP	FLT3	JUN	MLLT11	NTRK3	RET	SPTAN1	WAS
ABCC4	BLM	CLTC	ERBB3	FLT4	KDM5C	MLLT4	PAFAH1B2	RHOH	SRC	WRN
ABCG2	BMPR1A	CLTCL1	ERBB4	FOXA1	KDM6A	MMP11	PAK7	RICTOR	SRGAP3	WT1
ABI1	BMX	CNBP	ERC1	FOXL2	KDR	MMP14	PALB2	RIPK1	STIL	WTX
ABL1	BNC2	COL1A1	ERCC2	FOXO1	KDSR	MMP2	PARK2	RIPK2	STK11	XPA
ABL2	BRAF	COPEB	ERCC3	FOXO3	KEAP1	MMP24	PATZ1	RIPK3	SUFU	XPC
ACSL3	BRCA1	CRCT1	ERCC4	FOXO4	KIAA0774	MMP26	PAX5	RIPK4	SULT1A1	XPO1
ACSL6	BRCA2	CREBBP	ERCC5	FSCB	KIAA1303	MMP27	PBRM1	RNF213	SYK	YAP1
ACVR1B	BRIP1	CRKL	ERCC6	FUBP1	KIAA1549	MMP28	PDGFC	ROBO1	SYNE1	ZBED1
ADAMTSL3	BUB1	CRLF2	ERG	FYN	KIT	MMP8	PDGFRA	ROBO2	TARBP2	ZBED2
AFAP1	BUB1B	CRTC1	ESR1	GATA1	KLF6	MNX1	PDGFRB	ROS1	TBC1D29	ZBED3
AFF1	C1orf144	CSF1R	ESR2	GATA2	KRAS	MPL	PDPK1	RPN1	TBK1	ZBED4
AFF3	CACNA1B	CTNNA1	ESRP1	GATA3	LAMA5	MRE11A	PER1	rptor	TBX22	ZBTB16
AFF4	CAMK1D	CTNNB1	ETV1	GATA4	LCK	MSH2	PFAS	RUNX1	TCF1	ZFP36L1
AGTR1	CARD11	CUBN	ETV4	GATA6	LIFR	MSH6	PHF6	RUNX1T1	TCF4	ZIC4
AGTRAP	CARS	CXCR7	ETV5	GMPS	LMO1	MSN	PHOX2B	RUNX3	TCF7L2	ZMYM2
AKT1	CASC5	CYLD	ETV6	GNA11	LPHN2	MTHFR	PIK3CA	SBDS	TEC	ZNF668
AKT2	CAV1	CYP1B1	EWSR1	GNAQ	LPHN3	MTOR	PIK3CG	SDH5	TERT	TMPRSS2*
AKT3	CBFB	CYP2C19	EXT1	GNAS	LRP1B	MTUS2	PIK3R1	SDHAF2	TET1	
ALK	CBL	CYP2C8	EXT2	GOPC	LRP2	MUC2	PIM1	SDHB	TET2	
ALOX12B	CBLB	CYP2D6	EZH2	GPC3	LYN	MUTYH	PKDCC	SDHC	TET3	
ANXA7	CBLC	CYP3A4	FAM123B	GRAF	MADH4	MYC	PKHD1	SDHD	TFDP1	
APC	CCDC132	CYP3A5	FAM46C	GSTP1	MAGI1	MYCL1	PLA2G2D	SEC31A	TFG	
AR	CCDC6	DAXX	FANCA	GUCY1A2	MAGI2	MYCN	PML	SEPT5	TFRC	
ARAF	CCND1	DBN1	FANCB	HGF	MAGI3	MYD88	PMS1	SEPT9	TGFBR2	
ARHGAP26	CCND2	DDB2	FANCC	HIF1A	MALT1	MYH1	PMS2	SETD2	TIAM1	
ARID1A	CCND3	DDIT3	FANCD2	HIP1	MAML2	MYH11	PNRC1	SF3B1	TNFAIP3	
ARID2	CCNE1	DDR1	FANCE	HLA-A	MAN1B1	MYH9	PPARG	SGK1	TNFRSF11A	
ARID5B	CCNL1	DDR2	FANCF	HLA-DRB1	MAP2K1	MYST3	PPP2R1A	SGK3	TNFRSF14	

ARNT	*CD79A*	*DDX5*	*FANCG*	*HNF1A*	*MAP2K2*	*NBN*	*PRDM1*	*SH2B3*	*TNFRSF6*
ASCL1	*CD79B*	*DDX6*	*FANCI*	*HNF1B*	*MAP2K4*	*NBS1*	*PREX2*	*SH3GL1*	*TOP1*
ASXL1	*CDC42EP1*	*DICER1*	*FANCL*	*HRAS*	*MAP3K1*	*NCKIPSD*	*PRF1*	*SIL1*	*TP53*
ATIC	*CDC73*	*DIS3*	*FANCM*	*HRPT2*	*MAP3K5*	*NF1*	*PRKAR1A*	*SIX4*	*TP63*
ATM	*CDH1*	*DLC1*	*FAS*	*HSP90AA1*	*MAP3K7*	*NF2*	*PRKCI*	*SLC19A1*	*TP73*
ATP8B1	*CDH2*	*DNMT1*	*FBXO31*	*HSP90AB1*	*MAP3K8*	*NFE2L2*	*PRKDC*	*SLC22A18*	*TPM4*
ATR	*CDK12*	*DNMT3A*	*FBXW7*	*IDH1*	*MAP3K9*	*NFKB1*	*PRRX1*	*SLC22A2*	*TPMT*
ATRX	*CDK4*	*DOT1L*	*FCGR3A*	*IDH2*	*MAPK1*	*NFKBIA*	*PSIP1*	*SLC8A1*	*TRAF2*
AURKA	*CDK6*	*DPYD*	*FCHSD1*	*IGF1R*	*MAPK3*	*NKX2-1*	*PTCH1*	*SLCO1B3*	*TRAF5*
AURKB	*CDK8*	*EGFR*	*FCRL4*	*IGF2*	*MCL1*	*NLRP2*	*PTEN*	*SMAD2*	*TRIM24*
AURKC	*CDKN1A*	*EP300*	*FES*	*IKBKE*	*MDM2*	*NOTCH1*	*PTK2B*	*SMAD3*	*TRIM62*
AXIN1	*CDKN1B*	*EPHA1*	*FGFR1*	*IKZF1*	*MDM4*	*NOTCH2*	*PTPN11*	*SMAD4*	*TSC1*
AXIN2	*CDKN2A*	*EPHA3*	*FGFR2*	*IL21R*	*Mecon*	*NOTCH3*	*PTPRD*	*SMARCA4*	*TSC2*
AXL	*CDKN2B*	*EPHA4*	*FGFR3*	*IL6ST*	*MED12*	*NOTCH4*	*RABEP1*	*SMARCB1*	*TSHR*
BAP1	*CDKN2C*	*EPHA5*	*FGFR4*	*IL7R*	*MEN1*	*NPM1*	*RAD51L1*	*SMO*	*TTLL7*
BCL2	*CDX1*	*EPHA7*	*FH*	*INPP4B*	*MET*	*NQO1*	*RAF1*	*SOCS1*	*TYMS*
BCL2L1	*CEBPA*	*EPHA8*	*FHIT*	*ITK*	*MITF*	*NRAS*	*RANBP2*	*SOD2*	*UGT1A1*
BCL6	*CHEK1*	*EPHB1*	*FKBP9*	*ITPA*	*MLH1*	*NRP2*	*RARA*	*SOX2*	*UMPS*
BCR	*CHEK2*	*EPHB4*	*FLCN*	*JAK1*	*MLL1*	*NSD1*	*RB1*	*SP3*	*VHL*
BHD	*CHUK*	*EPHB6*	*FLNB*	*JAK2*	*MLL2*	*NTRK1*	*RECQL4*	*SPARC*	*VTI1A*

Genes in grey fields were tested for both somatic mutations and rearrangements.

**TMPRSS2* genes were only tested for rearrangements.

Supplementary Table S2. Clinicopathologic and follow-up information for the 15 study patients in AMC cohort

No.	Age /Sex	Primary site	Size (cm)	Function	PASS	Time to metastasis (years)	Familial	Metastatic site	Treatment	RECIST	survival (years)
#1	47/F	Uni PCC	3.2	Y	4	3.4	NA	kidney, peritoneal/ retroperitoneal cavity, LN (paraaortic)	Ex, Meta, RTx	CR	24.6 Alive
#2	30/F	PG[a]	12.5	N	6	4.3	NA	locoregional, bone	Ex, Meta, CTx, RTx	PD	15.6
#3	37/M	PG[b]	NA	Y	NA	0	NA	locoregional, bone, orbit	Ex, Meta, MIBG, CTx	PD	12.7
#4	69/F	Uni PCC	8	Y	5	6	NA	lung, liver	Ex	PD	7.2
#5	43/F	Bi PCC	NA	Y	NA	2.7	NA	liver	Ex, Meta, MIBG, CTx	PD	7.3
#6	34/F	Bi PCC	4.5	Y	6	8	(-)	LN (paraaortic)	Ex, Meta	CR	14.8 Alive
#7	49/F	Uni PCC	9.5	Y	11	1.4	NA	lung, bone	Ex, Meta, MIBG, CTx, RTx	PD	5.5
#8	65/F	Uni PCC	10	Y	8	6.3	(-)	LN (paraaortic)	Ex, Meta	SD	6.8 Alive
#9	18/M	Uni PCC	15	Y	13	0	NA	liver, lung	Ex, Meta, CTx	PD	0.6
#10	30/F	PG[a]	NA	Y	NA	4.1	NA	spinal cord, lung, bone	Ex, Meta, MIBG, RTx	PD	6.2
#11	49/F	Uni PCC	4.5	Y	5	0	(-)	bone	Ex, MIBG	PD	10.6 Alive
#12	34/F	Ubi PCC	NA	Y	NA	3.6	NA	liver	Ex, Meta, MIBG	SD	20 Alive
#13	44/F	PG[a]	7.1	Y	NA	6.5	NA	lung, bone, liver	Ex, Meta	SD	6.6
#14	15/M	Uni PCC	6.6	NA	NA	6.6	(-)	LN (retrocaval)	Ex, Meta	CR	15.7 Alive
#15	15/F	Uni PCC	6	Y	NA	7.1	RET	liver	Ex, Meta, TAE, MIBG	SD	15.8 Alive

[a]Infradiaphragm paraaortic PG; [b]Bladder PG; NA, not available; Y, Yes; N, No; PASS, Pheochromocytoma of the adrenal gland scaled score; Uni, unilateral; Bi, bilateral; LN, lymph node; Ex, excision of primary tumor; Meta, metastasectomy; CTx, chemotherapy; RTx, radiotherapy; MIBG, ^{131}I-meta-iodo-benzyl-guanidine; TAE, trans-arterial chemoembolization; RECIST, response evaluation criteria in solid tumors; CR, complete response; PR, partial response; SD, stable disease; PD, progressive disease.

Supplementary Table S3. Abbreviations for tumors and median tumor mutation burden of various cancer types

	cohort	Median	Full_Name
1	PPGL_AMC	0.58	Pheochromocytoma and Paraganglioma
2	PPGL_TCGA	0.18	Pheochromocytoma and Paraganglioma
3	THCA	0.2	Thyroid carcinoma
4	UVM	0.24	Uveal Melanoma
5	LAML	0.26	Acute Myeloid Leukemia
6	TGCT	0.28	Testicular Germ Cell Tumors
7	THYM	0.28	Thymoma
8	PPGL_TCGA_ aggressive	0.35	Pheochromocytoma and Paraganglioma
9	KICH	0.4	Kidney Chromophobe
10	PRAD	0.52	Prostate adenocarcinoma
11	ACC	0.54	Adrenocortical carcinoma
12	MESO	0.55	Mesothelioma
13	LGG	0.56	Brain Low Grade Glioma
14	PAAD	0.68	Pancreatic adenocarcinoma
15	CHOL	0.73	Cholangiocarcinoma
16	BRCA	0.78	Breast invasive carcinoma
17	SARC	0.82	Sarcoma
18	UCS	0.94	Uterine Carcinosarcoma
19	GBM	1.04	Glioblastoma multiforme
20	KIRC	1.04	Kidney renal clear cell carcinoma
21	KIRP	1.24	Kidney renal papillary cell carcinoma
22	OV	1.36	Ovarian serous cystadenocarcinoma
23	UCEC	1.47	Uterine Corpus Endometrial Carcinoma
24	LIHC	1.62	Liver hepatocellular carcinoma
25	CESC	1.72	Cervical squamous cell carcinoma and endocervical adenocarcinoma
26	READ	1.82	Rectum adenocarcinoma
27	HNSC	2.04	Head and Neck squamous cell carcinoma
28	ESCA	2.09	Esophageal carcinoma
29	STAD	2.23	Stomach adenocarcinoma
30	DLBC	2.26	Lymphoid Neoplasm Diffuse Large B-cell Lymphoma
31	COAD	2.28	Colon adenocarcinoma
32	BLCA	3.32	Bladder Urothelial Carcinoma
33	LUAD	3.78	Lung adenocarcinoma
34	LUSC	4.38	Lung squamous cell carcinoma
35	SKCM	8.29	Skin Cutaneous Melanoma

Supplementary Table S4. Known PPGLs-Related Genes not Included in the Targeted Next-Generation Sequencing Gene

Gene	*MAX, SDHA, TMEM127, EGLN1/PHD2, EPAS1, KIF1B, EGLN2/PHD1, H3F3A, KMT2D, MDH2, MERTK*
